# Direct Type I IFN but Not MDA5/TLR3 Activation of Dendritic Cells Is Required for Maturation and Metabolic Shift to Glycolysis after Poly IC Stimulation

**DOI:** 10.1371/journal.pbio.1001759

**Published:** 2014-01-07

**Authors:** Austin Pantel, Angela Teixeira, Elias Haddad, Elizabeth G. Wood, Ralph M. Steinman, M. Paula Longhi

**Affiliations:** 1 Laboratory of Cellular Physiology and Immunology and Christopher H. Browne Center for Immunology and Immune Diseases, The Rockefeller University, New York, New York, United States of America; 2 Vaccine and Gene Therapy Institute of Florida, Port St. Lucie, Florida, United States of America; 3 William Harvey Research Institute, Barts and The London School of Medicine and Dentistry, Queen Mary, University of London, London, United Kingdom; University of Pennsylvania, United States of America

## Abstract

Type I interferons (IFNs) play an important role in direct antiviral defense as well as linking the innate and adaptive immune responses. On dendritic cells (DCs), IFNs facilitate their activation and contribute to CD8^+^ and CD4^+^ T cell priming. However, the precise molecular mechanism by which IFNs regulate maturation and immunogenicity of DCs in vivo has not been studied in depth. Here we show that, after in vivo stimulation with the TLR ligand poly IC, IFNs dominate transcriptional changes in DCs. In contrast to direct TLR3/mda5 signaling, IFNs are required for upregulation of all pathways associated with DC immunogenicity. In addition, metabolic pathways, particularly the switch from oxidative phosphorylation to glycolysis, are also regulated by IFNs and required for DC maturation. These data provide evidence for a metabolic reprogramming concomitant with DC maturation and offer a novel mechanism by which IFNs modulate DC maturation.

## Abstract 2

Type I IFN signaling is indispensable for the maturation of dendritic cells (DCs) that are required to elicit an immune response, and it also controls a shift in cellular metabolism to meet the increased energy demands of DC maturation.

## Author Summary

Immune responses are orchestrated by a specialized cell type called dendritic cells (DCs). In order to achieve durable and robust immunity, DCs need to undergo an intricate differentiation process known as maturation. This process is poorly understood at the moment. Poly IC, which mimics viral RNA and is an agonist for the viral pattern recognition receptors (PRRs) that signal “danger” to the immune system, can induce full maturation of DCs and it has been shown that this requires type I IFN signaling. In this study we set out to examine the specific signals provided by direct PRR or IFN stimulation that are required for DC maturation. We found to our surprise that type I IFN can regulate almost all steps of the DC's maturation process without requiring direct PRR involvement. We also show that type I IFN regulates several metabolic switches essential for preservation of DC integrity: it stimulates the expression of the hypoxia-inducible factor 1α (Hif1α), which controls the metabolic shift from oxidative phosphorylation (used by resting cells to generate energy) to aerobic glycolysis, a less efficient but faster energy-producing process. This metabolic shift was required to meet increased energy demands of activated DCs and to prevent their premature death, thus sustaining an immune response to viral infection.

## Introduction

Dendritic cells (DCs) are the main antigen presenting cells for initiating primary immune responses [1]. DCs patrol the bloodstream and tissues to sense danger signals from bacteria, viruses, or toxins. After exposure to such stimuli they undergo an extensive maturation process, which results in the loss of phagocytic activity, expression of co-stimulatory molecules, cytokine production, and enhanced migration to draining lymph nodes, where they present antigens to naive T cells. This intricate differentiation process is essential for the induction of immunity, as DCs can generate tolerance in the absence of “danger” signals [2].

The finding that pro-inflammatory cytokines upregulate HLA and costimulatory molecules in vitro, led to the idea that a cytokine cocktail (e.g., TNF-α, IL-1β, IL-6, PGE_2_) could be used to mature DCs [3],[4]. However, these DCs failed to produce IL-12, exhibited weak immunogenicity, and thus are ineffective for clinical use [5],[6]. In contrast, DCs matured in response to TLR agonist were shown to unleash more potent immune responses. Reis e Sousa et al. [7],[8] demonstrated that a robust Th1 response required direct pattern recognition receptor (PRR) stimulation. We reported previously that the synthetic dsRNA poly IC, a TLR3 and MDA5 agonist, efficiently stimulates functional maturation of DCs and confers immunogenicity, and its effect was critically dependent on direct stimulation of the type I IFN receptor [9]. Inflammatory cytokines are known to provide a third signal for T cell activation [10]. However, little is known about their specific signals provided for DC maturation.

Type I IFNs are potent anti-viral cytokines. The absence of type I IFN signaling increases susceptibility to some virus infections as demonstrated in IFNAR^−/−^ mice and in humans who harbour natural mutations in the type I IFN signaling pathway [11],[12]. This increased susceptibility is partially due to the expression of interferon-stimulated genes (ISGs) that control viral infections through a diverse range of direct antiviral effector functions [13], but type I IFNs have also been implicated in the generation of adaptive immunity. IFNs are known to promote clonal expansion and survival of antigen-specific CD8^+^ T cells [14],[15] and to induce the maturation of dendritic cells, the critical antigen presenting cells for initiating immunity [9]. We and others have shown that IFNs promote the expression of co-stimulatory molecule, increase DC migration, and facilitate cross-priming of CD8^+^ T cells and antigen presentation to CD4^+^ T cells [9],[16]–[19]. While it has been established that type I IFNs play an important role in DC maturation, the biochemical pathways involved, and the exact contribution of IFNs during this process, remain poorly understood.

In this study, we set out to examine the specific signals provided by direct PRR or IFNs stimulation that are required for DC maturation. Using a mixed chimera model, we identified genes and biochemical pathways that were governed exclusively by IFNs and made several interesting findings. First, IFNs, and not PRR, dominate gene expression changes induced in response to poly IC stimulation. Second, under a normal inflammatory response and in the presence of type I IFN, direct PRR stimulation is not required for DC maturation. Third, IFNs control a wide variety of cellular processes. In addition to pathways associated to DC maturation, e.g., antigen processing, our study revealed an important role for IFNs in regulating several metabolic switches essential for preservation of cellular integrity. We show that direct IFNAR stimulation induces hypoxia-inducible factor 1 (Hif1α) expression, which is responsible for the metabolic transition from oxidative phosphorylation (OXPHOS) to glycolysis and the prevention of DCs premature cell death.

## Results

### Type I IFN Dominates the Transcriptional Response to Poly IC

**Figure 1:**
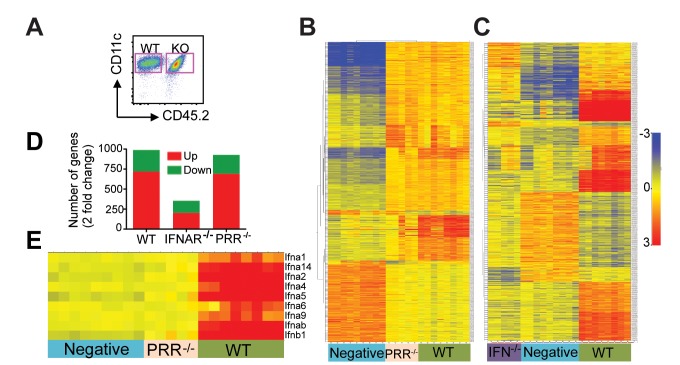
DCs transcriptional response to poly IC is highly dependent on type I IFNs. (A) Bone marrow mixed-chimera mice were injected with 50 µg poly IC IP. 4 h later, DCs were FACS sorted on the basis of CD45.2 expression and analyzed for gene expression with Illumina chips. Heat map represent expression values (2-fold change and FDR<0.05) of poly IC-treated WT (green), PRR^−/−^ (pink) versus untreated DCs (blue) (B) or WT, IFNAR^−/−^ (violet) versus untreated DCs (C). Each column represents an individual sample. The red and blue colors indicate high and low expression, respectively. (D) Similar results can be observed when depicted as bar graphs, where upregulated genes are shown in red and downregulated ones in green. (E) As in (B) but heat map represents expression values of genes belonging to type I IFN cytokines. Each column represents an individual sample. Graph represents the numbers of genes up- or downregulated in WT, IFNAR^−/−^, and PRR^−/−^ DCs after poly IC stimulation.

PRRs have long been thought to confer immunogenicity by inducing DC to undergo a series of phenotypic and functional changes that consequently result in T cell immunity. However, as demonstrated in our previous study, blocking type I IFN abolished DCs' immunogenicity conferred by poly IC stimulation [9]. This indicates that the intracellular events downstream of IFNAR, upon binding of type I IFN, differ from those downstream of PRR, and furthermore are indispensible for initiation of immune response. To differentiate the events directly resulting from IFNAR versus PRR on DCs in vivo, we generated mixed chimeric mice in which half of the bone marrow was derived from wild-type (WT) CD45.1 donors and the other half from PRR^−/−^ or IFNAR^−/−^ mice (CD45.2). Such system allows us to evaluate the intrinsic difference in DCs resulting from deficiency of respective receptor upon exposure to the same WT inflammatory environment. Mice were injected with 50 µg of poly IC intraperitoneally (IP) and 4 h later, splenic DCs were harvested, sorted on the basis of the expression of CD45.2 marker, and analyzed for gene expression by microarray (Figure 1A). Two types of negative controls were included. First, mixed chimera mice were injected with PBS to evaluate WT versus knockout (KO) DC development and maturation in the steady state. Second, PRR^−/−^ (MDA5/TLR3 double KO, which are the receptors for poly IC) mice were stimulated with poly IC to evaluate whether impurities or other substances in the adjuvant preparation could result in stimulation through pathways independent of PRR. No statistically significant gene-expression changes were detected between WT and KO DCs in steady state (PBS DCs) or poly IC-stimulated DCs from PRR^−/−^ mice. Similarly no differences were observed when compared to poly IC-stimulated DCs with single or pooled negative controls (Tables S1 and S2). Thus all negative controls were pooled. Poly IC treatment led to change of 988 genes in WT DCs (2-fold change and false discovery rate [FDR]<0.05) (Figure 1B and 1D). As early as 4 h post-stimulation, more than 700 genes were upregulated, whereas only 269 genes were downregulated. Strikingly, the majority of these changes were dependent on type I IFN and not PRR stimulation (Figure 1B–1C). PRR^−/−^ DCs showed a similar profile than WT DCs except for the expression of 26 genes, including all type I IFN cytokine genes that required direct PRR stimulation in order to induce their expression (Figure 1E; Table S1). In contrast, only 354 genes changed their expression in IFNAR^−/−^ DCs after poly IC (Figure 1D). These results confirm the importance of IFNs in controlling the process of DC maturation.

### Type I IFN Controls the Activation of Pathways Associated with DC Maturation

**Figure 2:**
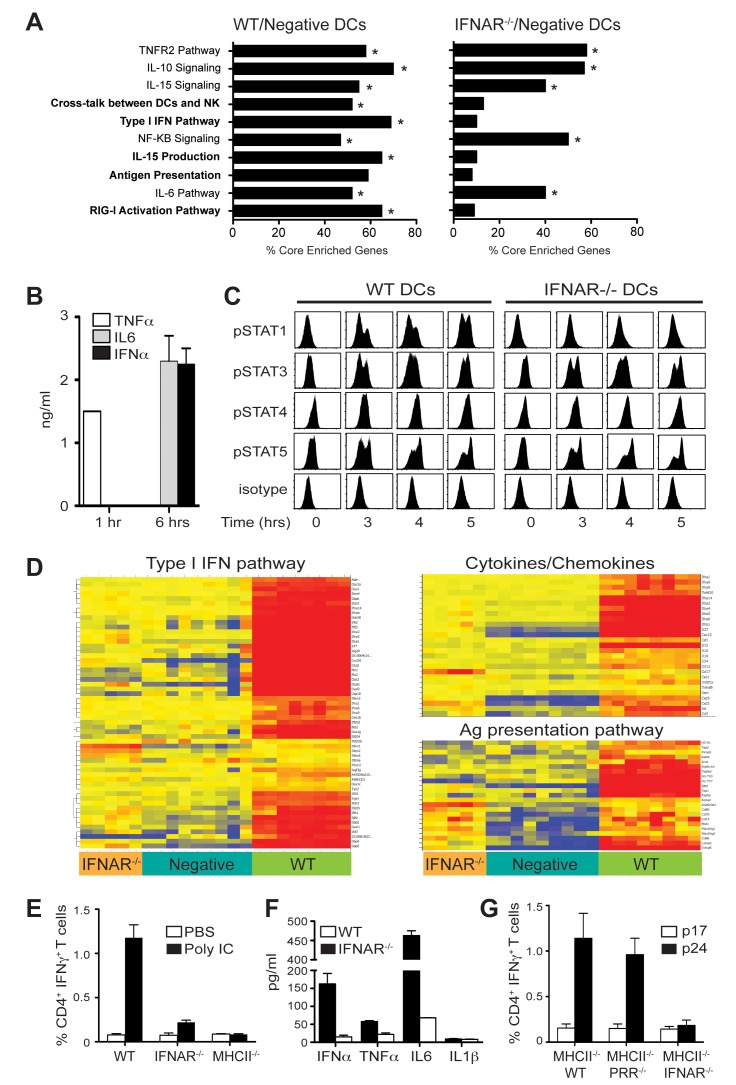
Impaired antigen presentation by IFNAR^−/−^ DCs. Bone marrow mixed-chimera mice were injected with 50 µg poly IC IP. 4 h later, purified DCs were analyzed for gene expression with Illumina chips. (A) Pathway analysis was performed using GSEA. Graph represents the top ten pathways upregulated in WT DCs after poly IC stimulation (FDR<0.25) and the correspondent pathways in IFNAR^−/−^ DCs. Bars indicate the percent of core enrichment genes for each set (defined as those genes ranked before or at the peak of the enrichment score). Significant pathways (FDR<0.25) are indicated with *. (B) WT/IFNAR^−/−^ mixed chimera mice were treated IP with 50 µg poly IC. At different time points, IL-6, TNF-α, and IFN-α were analyzed in serum. Error bars indicate the mean ± SD (*n* = 4). (C) Mixed chimera mice were treated IP with 50 µg poly IC. At different time points, activation of STATs transcriptional factors was evaluated by intracellular staining of the phosphorylated active form by Phospho Flow. Histograms are representative of three independent experiments. (D) Heat maps represent expression values of genes belonging to the type I IFN, cytokines/chemokines, and antigen presentation pathways. Each column represents an individual sample (six mice pooled). WT DCs are indicated in green, IFNAR^−/−^ in orange, and untreated DCs in blue. (E) Poly IC-treated WT, IFNAR^−/−^, and MHCII^−/−^ DCs were pulsed in vivo with DEC-p24 antigen and adoptively transferred into naïve mice. Antigen-specific response was evaluated by intracellular cytokines after prime-boost. Graph represents the percentage of CD3^+^ CD4^+^ T cells producing IFN-γ. Means ± SD are shown of three independent experiments with a total of nine mice. (F) WT/IFNAR^−/−^ mixed-chimera mice were stimulated with poly IC in vivo. 4 h later, WT and IFNAR^−/−^ DCs were purified by cell sort and plated at 1×10^5^ cells/well in a round-bottomed 96 well. Supernatants were collected after 18 h and cytokine production was evaluated by multiplex ELISA. Bars represent the mean ± SD from three independent experiments. (G) Mixed-chimera mice were vaccinated with 5 µg α-DEC-p24 along with 50 µg poly IC and 25 µg of αCD40 mAb IP Two weeks later, IFN-γ secretion in gated CD3^+^ CD4^+^ T cells from spleen, was measured in response to gag p24 peptide mix or control gag p17 peptide mix. Bars represent the mean ± SD from two experiments with six mice total.

Besides their well-known anti-viral function, it is increasingly recognized that IFNs contribute to DC maturation. The dramatic change in transcriptional profile downstream of IFNAR suggests that IFNs affect molecular pathways that lead to immunogenicity. To identify these pathways, we performed pathway analysis of the transcriptional changes using gene set enrichment analysis (GSEA) (FDR<0.25) [20]. Our analysis reveals that in response to poly IC treatment, both WT and IFNAR^−/−^ DCs activated pathways associated with general inflammation including NFκβ, IL6, IL-15, and IL10, indicating that stimulation from the inflammatory milieu is independent of IFNAR (Figure 2A; Table S2). To corroborate these findings, we first documented the systemic release of inflammatory cytokines in mixed chimeras. After poly IC injection, early production of TNF-α at 1 h and later release of IL-6 and IFN-α at 6 h post injection were detected in mixed-chimera mice as previously described for WT mice (Figure 2B) [9], indicating that both DCs were exposed to same inflammatory milieu. To prove cytokine stimulation of DCs, we evaluated the activation of STAT (p-STAT) transcriptional factor by Phosphoflow analysis. As expected p-STAT3 and p-STAT5, the signal transducers for IL-6, IL-10, and IL-15 pathway among others, were detected in WT as well IFNAR^−/−^ DCs, while only WT DCs showed STAT1 phosphorylation, the signature transducer for type I IFN pathway (Figure 2C). Activation of STAT4, the transducer involved in IL-12 pathway was not detected in either WT or IFNAR^−/−^ DCs, consistent with the absence of IL12p70 production by splenic DCs in response to poly IC as previously described [9].

In stark contrast, IFNAR^−/−^ DCs failed to increase expression of RIG-like helicase as well as other genes in the type I IFN pathway, including genes encoding proteins implicated in the major antiviral pathways such as the PKR and IFIT proteins, the 2–5A synthetase system (OAS2/3), and the Mx pathway (MX1/2) (Table S2) [13]. Interestingly, in comparison to WT, INFAR^−/−^ DCs failed to activate many genes directly associated with DC immunogenicity. These include genes involved in pathways of antigen processing and presentation such as *MHCII*, *tap*, *lamp2*, and DC-natural killer (NK) cross-talk, and IL15 production such as *Il18* and *Il15* (Figure 2A and 2D; and Table S2). To confirm this result, we studied HIV gag-specific immune priming in vivo. Mixed-chimera mice were immunized with anti-DEC-HIV gag and either poly IC or PBS. Four hours later, splenic DCs were harvested, purified by cell sorting, and used for vaccination of naïve mice (intravenous [IV]). MHCII^−/−^ DCs were used as negative control. As expected only poly IC–treated WT, and not IFNAR^−/−^ or MHCII^−/−^ DCs, induced antigen-specific T cell responses (Figure 2E). In addition, IFNAR^−/−^ DCs, despite being stimulated by an inflammatory response in vivo, were unable to produce cytokines that play an important role in T cell priming and memory fate (Figure 2F). In summary, our data show that IFNAR signaling plays an indispensible role in inducing DC's immunogenicity after poly IC treatment and raises an important question. Is direct PRR stimulation required for DC immunogenicity? To answer this question we generated mixed chimeric mice in which half of the bone marrow was derived from MHCII^−/−^ donors and the other half from WT, PRR^−/−^, or IFNAR^−/−^ mice and analyzed gag-specific T cell responses after in vivo vaccination. Despite the requirement of PRR signaling for the production of type I IFNs (Figure 1E), under a fully induced inflammatory response, direct PRR stimulation was not required for DC maturation as previously believed (Figure 2G) [7]. In addition, no differences were observed in T cell proliferation and differentiation (unpublished data). IFN alpha A/D (universal type I IFN) as stand-alone adjuvant was unable to prime T cells (unpublished data) indicating that type I IFNs are essential but not sufficient to mature DCs and other factors are required.

### IFNs Are Required for DC's Metabolic Reprogramming

**Figure 3:**
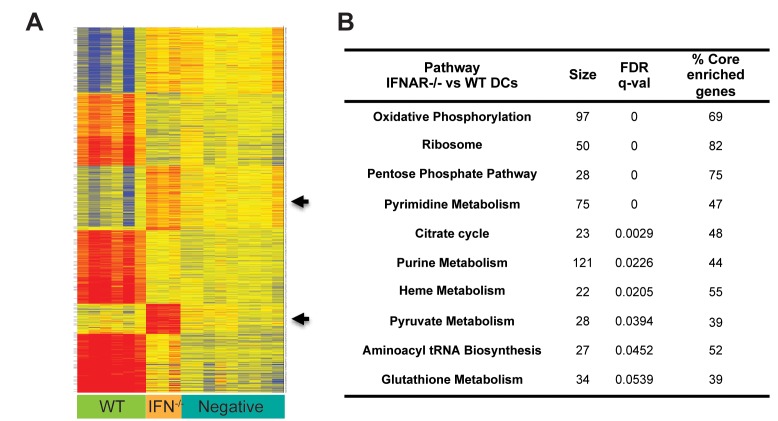
Altered metabolic response in IFNAR^−/−^ DCs. (A) Bone marrow mixed-chimera mice were injected with 50 µg poly IC IP. 14 h later, DCs were FACS sorted based on CD45.2 expression and analyzed for gene expression with Illumina chips. Heat map represents expression values (2-fold change and FDR<0.05) of poly IC-treated WT (green), IFNAR^−/−^ (orange) versus untreated DCs (blue). Each column represents an individual sample. (B) Pathway analysis was performed using GSEA (FDR<0.25). Table represents the top ten pathways upregulated in IFNAR^−/−^ versus WT DCs.

**Figure 4:**
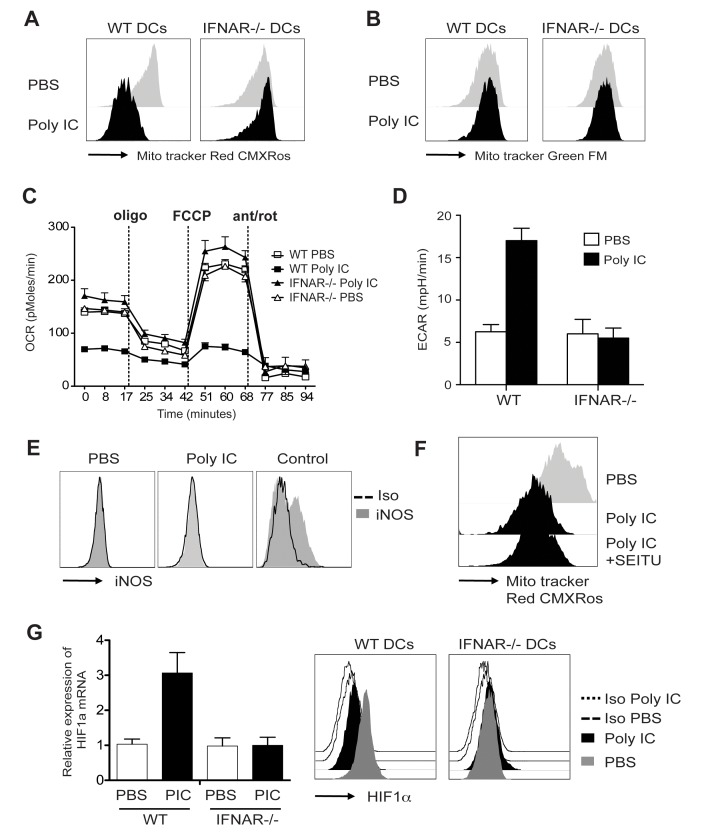
Changes in mitochondrial function after poly IC stimulation. WT/IFNAR bone marrow mixed-chimera mice were injected with 50 µg poly IC IP. 14 h later, spleen were harvested and incubated with the membrane potential-dependent stain MitoTracker Red CMXRos (A) or MitoTracker Green FM (B) for 20 min at 37°C. Cell staining was evaluated in CD11c^+^MHCII^+^B220^−^DX5^−^ DCs by flow cytometry. Histogram is representative of three independent experiments. (C) As (A) and (B) but after 14 h, CD11c^+^MHCII^+^B220^−^DX5^−^ DCs were sorted and seeded in a Seahorse XF-24 analyzer. OCR was determined in real time after the addition oligomycin, FCCP, and antimycin-A/rotenone. (D) ECAR values. Data represent means ± SD of quadruplicates. Data are representative of three experiments. (E) WT mice were injected with 50 µg poly IC IP 18 h later intracellular expressions of iNOS was evaluated by FACS. As positive control, monocyte-derived DCs were stimulated with LPS. Histogram are representative of three independent experiments. (F) As in (A), but WT were injected with 50 µg poly IC IP, spleen was harvested 4 h later and further incubated in the presence of SEITU ON. Histogram is representative of three independent experiments. (G) Chimera mice were stimulated with poly IC 4 h in vivo. Total RNA was isolated from sort-purified CD11c^+^MHCII^+^ DCs. Relative expression of HIF1α was analyzed by real time (RT)-PCR or by intracellular staining. mRNA levels in resting DCs were set to 1.

Our data revealed that over 900 genes changed their expression in response to poly IC in a type I IFN-dependent manner as early as 4 h. Similar findings were observed after 14 h stimulation when the majority of gene expression changes were regulated by IFNs (2-fold change and FDR<0.05) (Figure 3A). Surprisingly, a set of genes that were absent in WT DCs, were found upregulated in IFNAR^−/−^ DCs in response to inflammation after 14 h (Figure 3A, arrows). To understand this group of genes that appear to be suppressed by IFNAR signaling, we categorize these genes and biochemical pathways using GSEA. Our analysis revealed that the top pathways unregulated in IFNAR^−/−^ were associated with cell metabolism, e.g., OXPHOS (Figure 3B; Table S3), which was also evident after 4 h. Consistent with enhanced OXPHOS and mitochondrial respiration, mitochondrial membrane potential, an indicator of electron transport chain use, was significantly increased in IFNAR^−/−^ versus WT DCs after poly IC stimulation (Figure 4A) while mitochondrial mass was unchanged (Figure 4B). To confirm the decline in mitochondrial activity we used metabolic-flux analysis (Seahorse XF Analyser) to assess mitochondrial respiration by measuring oxygen-consumption rate (OCR) under basal conditions and after drug-induced mitochondrial stress [21]. Basal OCR was dramatically reduced in WT DCs after poly IC stimulation compared to untreated DCs. In contrast, there was no significant difference between PBS and poly IC-treated IFNAR^−/−^ DCs (Figure 4C). The sequential addition of the mitochondrial ATP-synthase inhibitor (oligomycin), the mitochondrial uncoupler FCCP, and the mitochondrial complex inhibitors antimycin-A and rotenone rotenone induced a characteristic OCR “drift” over time in steady state WT and IFNAR^−/−^ DCs, while OCR values were unchanged in poly IC-activated WT DCs (Figure 4C). In stark contrast, PBS and poly IC-treated IFNAR^−/−^ DCs showed similar changes in OCR values in response to pharmacological perturbation of mitochondrial function (Figure 4C). To determine weather reduced mitochondrial respiration in poly IC-stimulated DCs is a result of a metabolic shift to glycolysis, we analyzed the extracellular acidification rate (ECAR) as an indicator of lactate production and glycolysis. Poly IC stimulation of WT DCs resulted in an increase in glycolytic rate. However and consistent with OCR, ECAR values were similar between treated and un-treated IFNAR^−/−^ DCs (Figure 4D).

This finding indicates that DC activation with poly IC induces several metabolic changes most likely to preserve cell integrity while supporting the bioenergetically demanding processes of migration, cytokine secretion, and other effector functions and this process is regulated by type I IFNs. Recently, Pearce and colleagues showed that commitment to glycolysis on DCs was mediated by NO inhibition of OXPHOS [22]. However, this mechanism was limited to inflammatory monocyte-derived DCs and, consistent with their data, we could not detect iNOS production after poly IC stimulation or decreased mitochondrial membrane potential in the presence of the NOS inhibitor S-ethyl-isothiourea (SEITU) (Figure 4E and 4F). However, we could detect decreased expression of the Hif1 in IFNAR^−/−^ versus WT DCs (Figure 4G), which was shown to regulate commitment to glycolysis in other systems [23],[24]. These results show an association between IFNs and the control of metabolic pathways on DCs and suggest that this process can be regulated by Hif1α.

### Metabolic Switch to Glycolysis Is Regulated by Hif1α and Is Required for DC Immunogenicity

**Figure 5:**
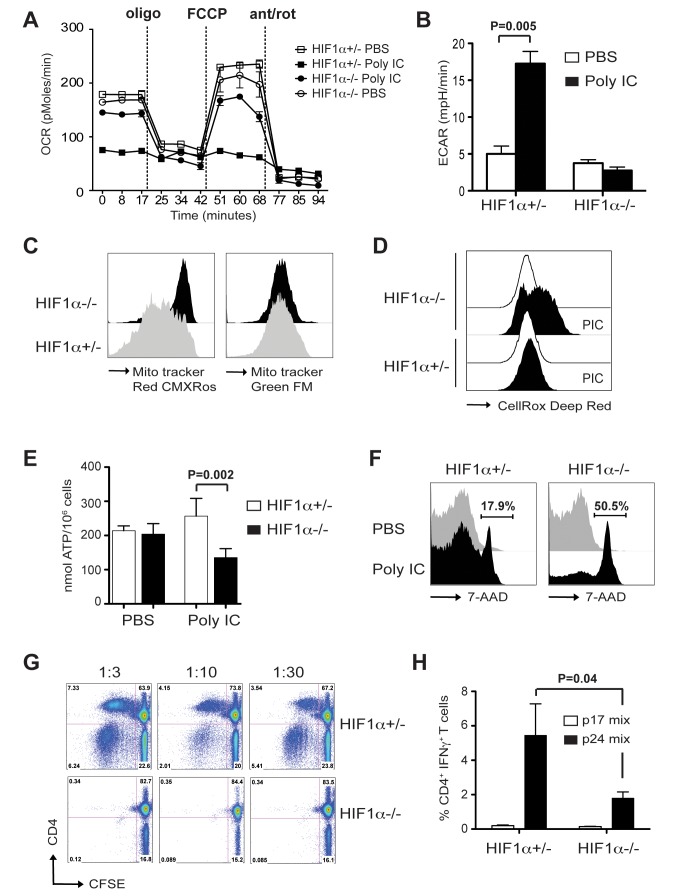
Hif1α is required for DC immunogenicity. Conditional Hif-1α^flox/−^. CD11c-Cre^+^ (HIF1α^−/−^) or HIF1α^+/−^ control mice were injected with 50 µg poly IC IP. 14 h later, spleen were harvested, CD11c^+^MHCII^+^B220^−^DX5^−^ DCs cell-sorted and seeded in a Seahorse XF-24 analyzer. (A) OCR in real time after sequential addition of oligomycin, FCCP, and antimycin-A/rotenone. (B) ECAR values. Data represent means ± SD of cuadriplicates. Data are representative of three experiments. (C) As in (A) but cells were incubated with MitoTracker Red CMXRos or MitoTracker Green FM and analyzed by flow cytometry. Histogram is representative of three independent experiments. (D) Hif1α^−/−^ and control mice were injected with 50 µg of poly IC and 14 h later splenic DCs were incubated with CellRox Deep Red reagent for 30 min at 37°C or (E) live purified CD11c^+^ MHCII^+^ DCs were lysed and ATP levels were measured by Elisa (*n* = 3). (F) Splenic DCs were stained with 7-AAD after 14 h stimulation with poly IC in vivo. Histogram is representative of three independent experiments. (G) HIF1α^−/−^ or HIF1α^+/−^ control mice were stimulated with 50 µg of poly IC. 18 h later, CD11c^+^ MHCII^+^ DCs were purified by cell-sort, fixed, and added in graded numbers to 2×10^5^ allogeneic Balb/C T cells. T cell proliferation was detected by CFSE dilution of CD3^+^ cells. Data are representative of three experiments. (H) Mice were primed and boosted 4 wk apart with 5 µg α-DEC-p24 and 50 µg poly IC. 1 wk later, IFN-γ secretion in gated CD3^+^ CD4^+^ T cells from spleen, was measured in response to gag p24 peptide mix or control gag p17 peptide mix. Bars represent the mean ± SD from two experiments with six mice total.

Hif1 functions as a master regulator of oxygen homeostasis, reduces oxygen consumption, and inhibits the generation of reactive oxygen species (ROS) from mitochondria, by switching glucose metabolism from OXPHOS to glycolysis. Hif1α is usually activated in response to hypoxia, but expression can be induced by TLR stimulation, though mechanism and consequence remain unclear [25]. To address the role of IFNAR signaling as a link between TLR stimuli, Hif1α upregulation, and metabolic reprogramming, we generated mice with conditional deletion of Hif1α in DCs by crossing Hif1α^flox/−^ with CD11c-Cre mice. Mice were healthy and showed no difference in DC numbers and activation markers in steady state compared to control DCs (unpublished data). We did not detect any difference between Hif1α^flox/flox^ and Hif-1α^+/−^, therefore Hif-1α^+/−^ mice were used as controls. Consistent with IFNAR^−/−^ DCs and supporting its role in controlling glycolytic switch, mitochondrial respiration, as assayed by real-time analysis of OCR, was restored in Hif1α^−/−^ DCs (Figure 5A). In addition, poly IC-stimulated Hif1α^−/−^ DCs displayed ECAR levels comparable to resting DCs (Figure 5B). Likewise, mitochondrial membrane potential, as well as total mitochondrial mass in Hif1α^−/−^ DCs, was unchanged after poly IC stimulation (Figure 5C). ROS are the natural by-products of mitochondrial respiration [26]; therefore, glycolytic switch may protect cells from oxidative stress and increase life span significantly. To test whether inhibition of Hif1α could result in high levels of ROS, we stained cells with CellROX Deep Red, a non-fluorescent reagent that becomes fluorescent upon oxidation by reactive oxygen. Relative to control DCs, Hif1α^−/−^ DCs demonstrated increased fluorescence staining after TLR stimulation (Figure 5D). To determine the impact of Hif1α knockdown on the cellular energy balance, ATP levels were measured after poly IC stimulation in vivo (Figure 5E). A modest increased in ATP levels were observed in WT DCs after poly IC stimulation, indicating that increased glycolytic rate was sufficient to sustain the ATP pool. In contrast, ATP levels were significantly lower in Hif1α^−/−^ DCs. ATP is necessary to maintain cell homeostasis and to promote cell survival since loss of intracellular ATP results in cell necrosis or apoptosis [27]. Indeed, we could detect increased cell death in Hif1α^−/−^, compared to WT DCs after poly IC stimulation (Figure 5F). Inhibition of NADPH oxidase by apocynin only partially decreased Hif1α^−/−^ and, to a lesser extent, WT DCs apoptosis (unpublished data) indicating that ROS levels are not the only reason for this effect. Together, this indicates that IFNAR signaling leads to upregulation of Hif1α, which may contribute to DC survival by suppressing production of reactive oxygen and maintenance of intracellular ATP levels.

To explore a causal link between IFNs, Hif1, DC survival, and immunogenicity, we assessed the ability of Hif1α^−/−^ DCs to induce T cell responses. To first test the functionality of DCs, we sorted CD11c^+^ MHCII^+^ DCs from spleen 18 h after poly IC injection and performed a mixed-leukocyte reaction (MLR). DCs from Hif1α^+/−^ but not Hif1α^flox/−^ CD11c-Cre^+^ mice became active stimulators of the allogeneic MLR, inducing robust proliferation of both CD4^+^ and CD8^+^ T cells (Figure 5G). Then, to evaluate the capacity of DCs to become immunogenic following antigen capture in vivo, we immunized mice with poly IC and Dec-HIVp24 targeted-vaccine, and analyzed immune T cell response after prime/boost. Consistent with the MLR, T cell response was reduced in Hif1α^flox/−^ CD11c-Cre^+^ compared to WT mice (Figure 5H), suggesting that increased cell death results in reduced T cell stimulation.

## Discussion

DC maturation is a highly complex differentiation process characterized by, but not limited to, upregulation of costimulatory molecules, production of inflammatory cytokines, downregulation of endocytic/phagocytic activity, changes in cell morphology and migration, and upregulation of the antigen processing machinery. Activation in *trans* by inflammatory mediators has been shown to induce phenotypically mature DCs. However, those DCs were unable to induce adaptive immunity. Increasing evidence suggest that direct pathogen stimulation via PRR, is required to induce fully mature DCs and to provide “signal, 3” e.g., cytokines for T cell priming [8],[28]. In our previous work, using poly IC as maturation stimuli, we found that direct PRR stimulation alone is insufficient to induce DC maturation and that immunogenicity requires direct signaling through type I IFN receptor [9]. In this work, we aimed to identify key biochemical pathways for DC maturation that were regulated by direct PRR or IFNAR stimulation. In order to do so, we performed gene array analysis from mixed chimera mice. In this model, DCs from WT bone marrow can respond directly to poly IC and type I IFNs and they mount a normal inflammatory response, whereas DCs derived from the KO bone marrow only respond to one receptor (PRR or IFNAR). However, both DCs receive the same secondary signals from the inflammatory environment (activation in *trans* due to poly IC response in WT radioresistant and radiosensitive cells). While, microarray analyses have been previously used to investigate the global effects of PRR and interferon stimulation in isolated cells in vitro [29]–[31], no information is currently available on genes directly regulated by PRR and type I IFNs in vivo, where inflammation is the consequence of the complex response of several cell populations to various stimuli.

The data presented in this paper show that bystander activation of PRR^−/−^ DCs induces similar gene expression profile to WT DCs, with the exception of type I IFN genes that required direct PRR engagement. However, in stark contrast from previous publications from Sporri and colleagues [28] and consistent with the microarray data, as long as type I IFNs were present in the inflammatory milieu, direct PRR stimulation was not required for DC immunogenicity. This difference could be attributed to the study of polyclonal T cell responses instead of TCR-transgenic models. This finding is of high relevance for the design of protein-based vaccine. The current paradigm is that adjuvant should be designed to activate specific PRR receptors expressed selectively on different DC subsets. DC subsets express an array of different PRR, which will require a perfect matching of the adjuvant and the antigen-presenting cell. Our data refute this model and suggest that DCs can be activated *in trans* by different PRR agonist.

While direct PRR stimulation had little impact in the gene expression profile of poly IC-stimulated DCs, the overall response was dramatically reduced in IFNAR^−/−^ DCs. This is particularly surprising because poly IC induces high levels of pro-inflammatory cytokines, in particular TNF-α, which has been described to activate DCs [32],[33]; yet we found that pro-inflammatory cytokines or direct PRR engagement, in the absence of IFNAR signaling, were unable to upregulate pathways associated with DC maturation, including upregulation of costimulatory molecules, and were dispensable for DC immunogenicity. More strikingly, the data presented here indicate that type I IFNs can also regulate metabolic pathways. After a transient increase in mitochondrial activity, activated DCs switch from mitochondrial OXPHOS to aerobic glycolysis as the major source of ATP [22],[34]. Glycolysis generates ATP with lower efficiency but at a faster rate than OXPHOS [35]. We confirmed similar metabolic shifts in DCs in vivo. WT DCs showed an upregulation of Hif1α transcription followed by a decrease in mitochondrial respiration and increase in glycolytic rate. To our surprise, metabolic reprogramming was impaired in IFNAR^−/−^ DCs. IFNAR^−/−^ DCs utilize OXPHOS as the main source of ATP in an attempt to rebalance their energy supply via increased respiration, which was evident by upregulation of the OXPHOS, citrate cycle, and pyruvate pathways. One could argue that failure to switch to glycolysis is rather a consequence than the cause of DC inactivation. Our experimental approach relies on the use of mixed-chimeric mice where both DCs are exposed to the same inflammatory milieu. We could detect partial upregulation of costimulatory molecules as well as STAT3 and STAT5 phosphorylation indicating that IFNAR^−/−^ DCs were stimulated by other cytokines and inflammatory mediators. Furthermore, membrane potential was increased in poly IC-stimulated compared to steady state-IFNAR^−/−^ DCs showing that those DCs have an altered metabolic response to inflammation.

Metabolic reprogramming appears to prevent bioenergetic collapse in response to the initial increase in energy consumption associated with signal transduction and transcription. Recently, it has been recognized that T cell metabolism is regulated to support cell proliferation and control cell trafficking [36], but how it affects DC maturation is unclear. The transcription factor Hif1, which can be activated by low oxygen tensions or TLR stimulation, promotes the shift from oxidative to glycolytic metabolism [37]. LPS-stimulated mouse DCs under hypoxia increased expression of costimulatory molecules, proinflammatory cytokine, and induction of MLR. Inhibition of Hif1α expression with siRNA, significantly reduces glucose usage, inhibits DC maturation and MLR stimulation [38]. However, others have shown reduced migratory capacity and impaired DC maturation after exposure to low oxygen tension and Hif1 activation [39]–[42]. Experiments were performed in vitro with artificial culture conditions, instead of in vivo with genetic approaches, which may explain these contradictory results. Using conditional KO mice, we showed that Hif1α upregulation was required for metabolic switch to glycolysis as well as for DC survival and immunogenicity in vivo. During the revision process of this manuscript, two reports of the important role of Hif1α on DC's function were published [43],[44]. The authors had used a similar genetic targeting approach to delete expression of Hif1α and showed regulation of bone marrow-derived DC's cytokine expression and antigen presentation capacity. However, the exact mechanism and the association with cell metabolism were not investigated.

While the focus of this study was to define actions of PRR and IFNs in the activation of DCs, these data also helped to elucidate additional roles of interferons in cell metabolism. Exploration of the transcriptional program initiated by the adjuvant poly IC and controlled directly by IFNs, extends our understanding of the global actions of these cytokines and improves our comprehension of the many steps required for DC maturation.

## Materials and Methods

### Mice

C57BL/6, Hif1α^flox/flox^, and CD11c-Cre^+^ mice were purchased from Jackson Laboratory; IFNAR^−/−^, MDA5^−/−^, and MDA5xTLR3^−/−^ mice were kindly provided by K. Murali-Krishna (University of Washington, Seattle) and M. Colonna (Washington University, St. Louis, MO). To increase efficiency of CRE recombination, Hif1α^flox/flox^ mice were first crossed with EIIA-CRE to obtain Hif1α^flox/−^. Mice were then crossed with CD11c-Cre. Hif1α deletion was detected using the primers TTGGGGATGAAAACATCTGC and GCAGTTAAGAGCACTAGTTG. Mice were maintained under specific pathogen-free conditions and used at 7–8 wk of age according to Institutional Animal Care and Use guidelines.

### DC Innate Responses

Mice were injected IP with 50 µg of poly IC. For cytokine production, spleens were harvested 4 h after in vivo stimulation. CD11c^+^ MHCII^+^ DCs were purified by cell sorting (FACSAria; BD Biosciences) and plated at 5×10^4^ cells/well in a 96 well plate for 18 h prior to assay of cytokines in the supernatants by multiplex ELISA (R&D Systems; eBioscience). Alternatively, serum samples were collected 1 and 6 h after poly IC injection. For intracellular detection of phosphorylated STAT, purified CD11c^+^ MHCII^+^ DCs from mixed-chimera mice, were incubated in vitro with 25 µg/ml of poly IC and collected at different time points. For Hif1α expression, mixed-chimera mice were injected with 50 µg of poly IC for 4 h. Cells were fixed with 2% paraformaldehyde, permeabilized with cold 80% methanol and stained with PE- anti phospho Stat antibodies (BD Bioscience) or purified anti-Hif1α antibody (Novus Biologicals) followed by Alexa Fluor 555 goat anti rabbit secondary antibody (Invitrogen). Percentage of positive WT and IFNAR^−/−^ DCs was analyzed by flow cytometry.

### Metabolism Assays

Mitochondrial mass and potential were measured with MitoTracker Green FM and MitoTracker Red CMXRos (Invitrogen), respectively, by flow cytometry following manufacturers instructions. Oxidative stress was evaluated with CellRox Deep Red (Invitrogen) and flow cytometry according to the manufacturer's instructions. For ATP analysis, mice were injected with 50 µg of poly IC. After 14 h, live CD11c^hi^ CD3^−^ DX5^−^ B220^−^ DCs were cell-sorted, washed, and resuspended in 100 µl/10^6^ cells of PBS. Samples were boiled for 5 min and ATP concentration was measured with ATP determination kit (Invitrogen). For real-time analysis of the ECAR and the OCR, the Seahorse XF-24 metabolic extracellular flux analyzer was used (Seahorse Bioscience). In brief, mice were injected with PBS or 50 µg of poly IC. After 14 h, live CD11c^hi^ CD3^−^ DX5^−^ B220^−^ DCs were cell-sorted, washed, and resuspended in XF assay medium (unbuffered DMEM supplemented with 10 mM glucose, 2% FCS, 100 U/ml penicillin/streptomycin, 2 mM L-glutamine, and pyruvate) and plated onto Seahorse X24 cell plates at 4×10^5^/well. Perturbation of mitochondrial function was achieved by addition of 1 µM oligomycin, 1.5 µM fluoro-carbonyl cyanide phenylhydrazone (FCCP), and 100 nM rotenone plus 1 µM antimycin A (Sigma-Aldrich). Oligomycin is an ATP synthase inhibitor, FCCP a protonophore that uncouples ATP synthesis from the electron transport chain, rotenone and antimycin Aare complex I and III inhibitors, respectively. OCR was measured prior and after the addition of the indicated drugs per the manufacturer's instructions (Seahorse Bioscience).

### DC Antigen Presentation

To test allostimulatory capacity, spleen and node CD11c^+^ MHCII^+^ DCs were isolated 18 h after poly IC injection by cell sorting. DCs were fixed with 1% paraformaldehyde for 10 min on ice and added in graded numbers to 2×10^5^ CFSE-labeled (Molecular Probes) Balb/C T cells. After 5 d of culture, samples were stained with Live/Dead Fixable Violet viability dye (Invitrogen), AlexaFluor700-anti-CD3, PerCP-Cy5.5-anti-CD4, APC-eFluor780-anti-CD8, and acquired on a BD LSR II flow cytometer. For DC antigen presentation in vivo, chimera mice and MHCII^−/−^ mice as negative control, were injected with 5 µg of gag-p24 together with 50 µg of poly IC. After 4 h, splenic WT or IFNAR^−/−^ CD11c^+^ MHCII^+^ DCs were purified by cell sorting based on the expression of CD45.2 and adoptively transferred into naïve mice (IV). Alternatively, mice were immunized twice IP at 4 wk intervals with 5 µg of HIV gag-p24 plus 50 µg of poly IC. One week later, splenocytes were restimulated with p24 or p17 negative control peptide mix as previously described [9]. Antigen-specific responses were evaluated by intracellular IFN-γ after prime-boost.

### Microarray Analysis

Bone marrow mixed-chimera mice were injected with 50 µg poly IC IP 4 and 14 h later, WT and KO CD11c^hi^ CD3^−^ DX5^−^ B220^−^ DCs, were FACS sorted on the basis of CD45.2 expression. Total RNA was isolated using a Trizol (Invitrogen) and with RNeasy minelute cleanup kit (Qiagen). RNA was quantified using a Nanodrop 1000D spectrophotometer (ThermoScientific) and quality tested with Agilent 2100 Bioanalyser (Agilent Technologies). cRNA was labeled and hybridized onto mouseRef-8 Expression BeadChips v2.0 (Illumina) as per manufacturer's instructions and data were extracted using BeadStudio software (Illumina). At least triplicate samples were analyzed to achieve statistical significance. Raw data from Illumina single color chips were analyzed with GeneSpring 10.0. Intensities below background intensity were replaced with background intensity. The data were log transformed and the quantile normalization algorithm was applied. Data were filtered to eliminate probes with low expression intensity. Baseline transformed was performed to the median of all samples. Multiple t-tests were used to find differentially expressed genes (*p*-values adjusted with Benjamini-Hochberg method using FDR of 0.05). Differential expression was defined as 2-fold increase or decrease in poly IC stimulated WT, IFNAR^−/−^, or PRR^−/−^ over non-stimulated DCs. Significantly differentially regulated pathways were identified by GSEA (FDR of 0.25). The microarray data are available through the National Center for Biotechnology Information Gene Expression Omnibus (GEO) under accession number GSE46478.

### Real-Time PCR

Total RNA was isolated as described above. High-capacity RNA-to-cDNA Kit (Applied Biosystems) was used for cDNA reactions and stored at −80°C. Primers for HIF1α and GAPDH were selected and purchased from Applied Biosystems Gene Expression Assay Selection. GAPDH gene was used as a housekeeping gene to normalize results. Quantitative real-time PCRs were performed using TaqMan Universal PCR Master Mix in a 7900HT Real-Time PCR Instrument (Applied Biosystems).

### Statistical Analysis

Data reported in the figures represent the average of at least three independent experiments. Statistical significance was determined by Student's t-test with two-tailed *p*-values of 0.05 or less. Error bars represent the means ± standard deviation (SD). Data were analyzed and charts were generated using Prism 5 (GraphPad Software).

## Supporting Information

Table S1 **List of differentially expressed genes in PRR^−/−^ versus WT DCs after poly IC stimulation (4 h).** Fold changes are indicated as FC and p-values adjusted with Benjamini-Hochberg method as adj Pval.
(XLSX)

Table S2 **List of core enriched genes (GSEA) of all pathways in** **Figure 2** **.** Table displays the list of genes in each pathway that were core enriched in negative controls (pooled) compared to poly IC-treated WT DCs. Fold changes are indicated as FC and *p*-values adjusted with Benjamini-Hochberg method as adj Pval. In addition, the FC and Pval of genes when compared to steady state, single, negative controls, are also shown.
(XLSX)

Table S3 **Core enriched genes (GSEA) of all pathways in** **Figure 3** **.** Table displays the list of genes that were core enriched in each pathway after GSEA analysis and their fold change (FC) and *p*-value.
(XLSX)

## Abbreviations

DC: dendritic cell
ECAR: extracellular acidification rate
GSEA: gene set enrichment analysis
FDR: false discovery rate
IFN: interferon
IP: intraperitoneal
KO: knockout
MLR: mixed-leukocyte reaction
OCR: oxygen-consumption rate
OXPHOS: oxidative phosphorylation
PRR: pattern recognition receptor
ROS: reactive oxygen species
SD: standard deviation
WT: wild type
